# 3D Metrology Using One Camera with Rotating Anamorphic Lenses

**DOI:** 10.3390/s22218407

**Published:** 2022-11-01

**Authors:** Xiaobo Chen, Jinkai Zhang, Juntong Xi

**Affiliations:** 1 School of Mechanical Engineering, Shanghai Jiao Tong University, Shanghai 200240, China; 2 School of Mechanical Engineering, Jinan University, Jinan 250022, China

**Keywords:** 3D reconstruction, anamorphic lens, anamorphic stereo vision

## Abstract

In this paper, a novel 3D metrology method using one camera with rotating anamorphic lenses is presented based on the characteristics of double optical centers for anamorphic imaging. When the anamorphic lens rotates −90° around its optical axis, the 3D data of the measured object can be reconstructed from the two anamorphic images captured before and after the anamorphic rotation. The anamorphic lens imaging model and a polynomial anamorphic distortion model are firstly proposed. Then, a 3D reconstruction model using one camera with rotating anamorphic lenses is presented. Experiments were carried out to validate the proposed method and evaluate its measurement accuracy. Compared with stereo vision, the main advantage of the proposed 3D metrology approach is the simplicity of point matching, which makes it suitable for developing compact sensors for fast 3D measurements, such as car navigation applications.

## 1. Introduction

In modern industry, 3D metrology is an important technology, based on various methods. Optical metrology and non-optical metrology are the two categories of 3D metrology [1]. Non-optical 3D metrology methods may use a coordinate measuring machine (CMM), a scanning electron microscope, and a scanning probe microscope [2,3], whereas optical 3D metrology methods may employ chromatic confocal microscopy, point autofocus instruments, focus variation instruments, phase-shifting interferometry, coherence scanning interferometry, imaging confocal microscopy, and stereo vision. An overview of such methods was presented in detail [4,5,6]. Each 3D metrology method has its own properties and differs in application areas, measurement accuracy, scale, efficiency, cost, etc. Optical metrology procedures are often fast, precise, and non-destructive of the measured objects. Stereo vision, which uses two cameras with spherical lenses, is a frequently used optical metrology approach [7,8,9]. The measuring precision of stereo vision can range from several microns to several millimeters, depending on the measured area and the spherical lenses used. The most challenging aspect of stereo vision involves the extraction of the corresponding points from stereo images. Projectors are widely used to generate structured light to achieve high-quality corresponding points [10]. Stereo vision has been widely used in many areas, such as industry metrology, agriculture, daily living, etc. [11,12]

Anamorphic lenses are increasingly employed in the film industry to capture wide images for broad screens [13,14,15]. Anamorphic lenses are distinguished by two characteristics: double focal lengths and double optical centers [16,17,18]. Double focal lengths means the focal lengths differ greatly in the tangential and sagittal planes. This characteristic of anamorphic lenses is often used in the film industry, as it allows images to be compressed horizontally. Metrology, physics, and some optical imaging applications also use anamorphic lenses [19,20,21,22]. Double optical centers means there are two optical centers, one in the tangential plane and the other in the sagittal plane. The distance between the two optical centers is the anamorphic distance, which is an intrinsic quantity for anamorphic lenses. The anamorphic distance will provide anamorphic images with more 3D information, making anamorphic lenses considerably more suitable for 3D metrology. H. Durko used anamorphic lenses for measuring parts with large aspect ratios [23]. F. Blais performed an exploratory calibration of anamorphic lenses based on a pinhole imaging model [24]. In our previous work, a high-precision anamorphic lens calibration method was proposed with 3D and 2D calibration targets [16,25].

In this study, a novel 3D metrology method using one camera with rotating anamorphic lenses is proposed based on its characteristic of double optical centers. When the anamorphic lens rotates −90° around its optical axis, the 3D data of the measured object can be reconstructed from the two anamorphic images captured before and after the anamorphic rotation. The rest of this paper is organized as follows. Section 2 presents the anamorphic lens imaging model as well as the anamorphic distortion model. Section 3.1 describes the 3D reconstruction process employing rotating anamorphic lenses. Section 3.2 describes the point matching approach, and Section 3.3 depicts the rotating anamorphic stereo vision. Section 4 describes the experiments and evaluates the proposed method’s measurement accuracy. Finally, Section 5 concludes the paper.

## 2. Anamorphic Imaging Model

This section presents the imaging model and the distortion model of anamorphic lenses, which differ significantly from those based on spherical lenses. Anamorphic lenses are typically composed of rear spherical lenses on the back and an anamorphic attachment on the front. The anamorphic attachment consists of cylindrical lenses that have no optical power in planes parallel to the cylindrical axis but have optical power in planes perpendicular to it.

### 2.1. Anamorphic Imaging Model

**Figure 1:**
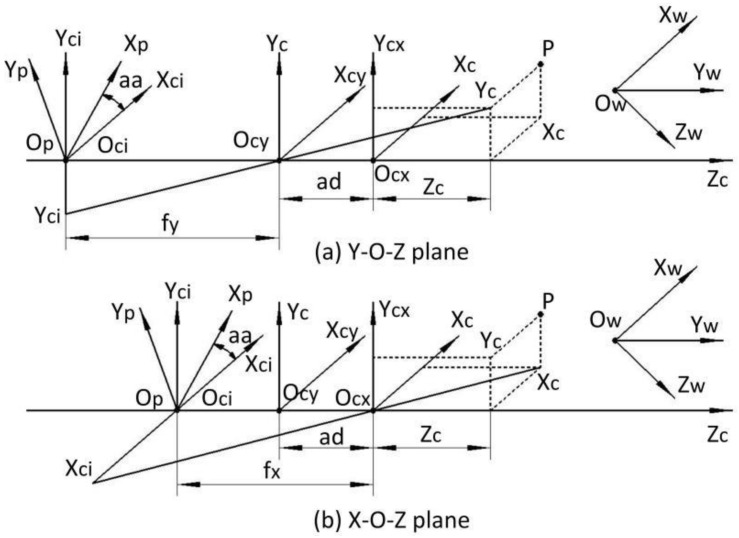
Anamorphic imaging model. (**a**) Imaging rays in the *Y_c_-O_cy_-Z_c_* plane; (**b**) imaging rays in the *X_c_-O_cx_-Z_c_* plane.

The imaging model of anamorphic lenses differs from the traditional pinhole imaging model of spherical lenses. The anamorphic distance and the anamorphic angle are introduced as new extra intrinsic parameters [16]. Figure 1 depicts the imaging model of anamorphic lenses, where *ad* denotes the anamorphic distance, and *aa* denotes the anamorphic angle. If the anamorphic lens is precisely assembled, the anamorphic angle *aa*, as shown in Figure 1, can be as small as zero. The cylindrical lenses or the anamorphic attachment determine the anamorphic distance *ad*, which must be precisely calibrated. *O_W_*-*X_W_Y_W_Z_W_* are the world coordinates, *O_ci_*-*X_ci_Y_ci_Z_c_* are the camera coordinates centered in the CCD plane, and *O_p_*-*X_p_Y_p_Z_c_* are the pixel coordinates centered in the CCD plane. *O_cx_* and *O_cy_* are the optical centers in the horizontal plane and the vertical plane, and *f_x_* and *f_y_* are the focal lengths in the two planes. The imaging model for anamorphic lenses can be expressed as follows:

(1) $$\begin{array}{l} \left[\begin{array}{c} X_{C}\\ Y_{C}\\ Z_{C} \end{array}\right]=\left[\begin{array}{cc} R & T \end{array}\right]\left[\begin{array}{c} X_{W}\\ Y_{W}\\ Z_{W}\\ 1 \end{array}\right]\\ \frac{Y_{I}}{f_{y}}=\frac{Y_{C}}{Z_{C}+ad}\\ \frac{X_{I}}{f_{x}}=\frac{X_{C}}{Z_{C}}\\ \left[\begin{array}{c} X_{P}\\ Y_{P} \end{array}\right]=\left[\begin{array}{cc} \cos\left(aa\right) & \sin\left(aa\right)\\ -\sin\left(aa\right) & \cos\left(aa\right) \end{array}\right]\left[\begin{array}{c} X_{I}\\ Y_{I} \end{array}\right] \end{array}$$

where [*X_w_*; *Y_w_*; *Z_w_*] is a point in the world coordinates, [*X_c_*; *Y_c_*; *Z_c_*] is the point expressed in the anamorphic coordinates. *R* and *T* denote the rotational and translating matrix from the world coordinates to the anamorphic coordinates. (*X_I_*, *Y_I_*) are the image coordinates in the camera coordinates in the image plane, and (*X_P_*, *Y_P_*) are the image coordinates in the pixel coordinates in the image plane.

### 2.2. Anamorphic Distortion Model

Anamorphic lenses have a distinct distortion model with respect to spherical lenses. The front anamorphic attachment introduces many more aberrations [26]. Based on the aberration theory and numerical experiments for anamorphic lenses, we proposed a polynomial distortion model for anamorphic lenses, which allows for high-precision camera calibration [25]. As shown in Equation (2), anamorphic distortions can be divided into radial distortions (*X_rad_*, *Y_rad_*), third-order distortions (*X*_3_, *Y*_3_), and second-order distortions (*X*_2_, *Y*_2_). (*x_d_*, *y_d_*) denotes the distorted image coordinates, and (*x_c_*, *y_c_*) denotes the undistorted image coordinates. [*k*_1_, *k*_2_, *n*_1_, *n*_2_, *m*_1_, *m*_2_, *x_2_*_1_, *x*_12_, *x*_03_, *x_2_*_0_, *x*_11_, *x*_02_, *y_3_*_0_, *y_2_*_1_, *y*_12_, *y*_03_, *y_2_*_0_, *y*_11_, *y*_02_] are the 19 distortion coefficients for anamorphic lenses that must be calibrated.

(2) $$\begin{array}{l} x_{c}=\underset{Xrad}{\underbrace{x_{d}\left(1+k_{1}\left(x_{d}^{2}+y_{d}^{2}\right)+k_{2}{\left(x_{d}^{2}+y_{d}^{2}\right)}^{2}+m_{1}\left(a^{2}x_{d}^{2}+y_{d}^{2}\right)+m_{2}{\left(a^{2}x_{d}^{2}+y_{d}^{2}\right)}^{2}\right)}}\\ +\underset{X_{3}}{\underbrace{x_{21}x_{d}^{2}y_{d}+x_{12}x_{d}y_{d}^{2}+x_{03}y_{d}^{3}}}\\ +\underset{X_{2}}{\underbrace{x_{20}x_{d}^{2}+x_{11}x_{d}y_{d}+x_{02}y_{d}^{2}}}\\ y_{c}=\underset{Yrad}{\underbrace{y_{d}\left(1+n_{1}\left(x_{d}^{2}+y_{d}^{2}\right)+n_{2}{\left(x_{d}^{2}+y_{d}^{2}\right)}^{2}+m_{1}\left(a^{2}x_{d}^{2}+y_{d}^{2}\right)+m_{2}{\left(a^{2}x_{d}^{2}+y_{d}^{2}\right)}^{2}\right)}}\\ +\underset{Y_{3}}{\underbrace{y_{30}x_{d}^{3}+y_{21}x_{d}^{2}y_{d}+y_{12}x_{d}y_{d}^{2}+y_{03}y_{d}^{3}}}\\ +\underset{Y_{2}}{\underbrace{y_{20}x_{d}^{2}+y_{11}x_{d}y_{d}+y_{02}y_{d}^{2}}} \end{array}$$

## 3. 3D Metrology Using Rotating Anamorphic Lenses

### 3.1. Description of 3D Metrology Using Rotating Anamorphic Lenses

**Figure 2:**
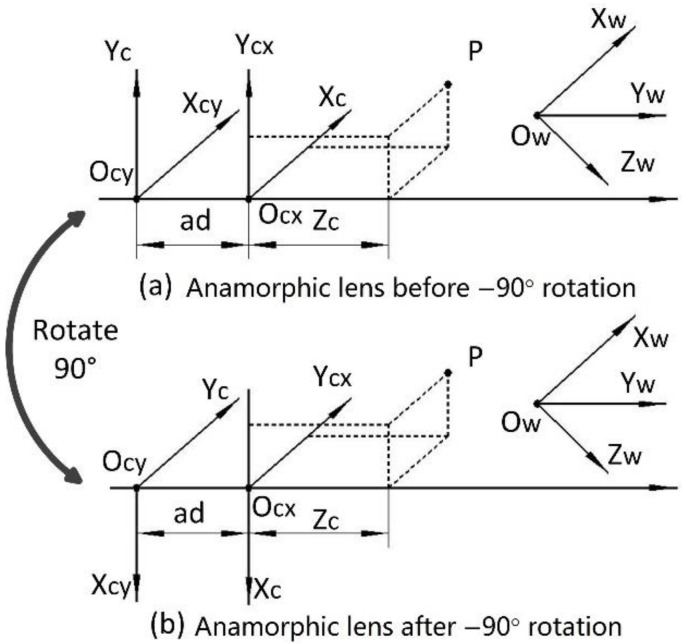
The two anamorphic positions. (**a**) Vertical position and (**b**) horizontal position which is achieved by a rotation of the anamorphic lens in the vertical position by −90°.

As shown in Figure 2a, the vertical position is the first anamorphic position, and the cylindrical axis of the anamorphic attachment is vertical. After a −90° rotation, the cylindrical axis of the anamorphic lens is horizontal, and this anamorphic position is known as the horizontal position. The anamorphic lens can switch between the two positions by rotating. The imaging model of anamorphic lenses shown in Section 2 can be reduced to an ideal anamorphic lens for ease of analysis, which implies that the anamorphic angle is zero, there are no distortions, and the rotation angle is precisely −90° without decentering. As shown in Figure 1 and Equation (1), the following equations describe the vertical position of an ideal anamorphic lens:

(3) $$\begin{array}{l} x_{v}=\frac{X_{C}}{Z_{C}}f_{x}\\ y_{v}=\frac{Y_{C}}{\left(Z_{C}+ad\right)}f_{y} \end{array}$$

where (*x_v_*, *y_v_*) refer to the image position of the object point *P_V_ =* (*X_C_*, *Y_C_*, *Z_C_*), and *P_V_* is in the anamorphic coordinates in the vertical position. After the anamorphic rotation, the coordinates of the object point in the anamorphic coordinates change as follows:

(4) $$P_{H}=RP_{V}$$

If the anamorphic lens rotates alone its optical axis by −90°, *R* can be expressed as:

(5) $$R=\left[\begin{array}{ccc} 0 & 1 & 0\\ -1 & 0 & 0\\ 0 & 0 & 1 \end{array}\right]$$

Thus, when the anamorphic lens is rotated to its horizontal position, the object coordinates can be calculated as *P_H_ =* [*Y_C_*; −*X_C_*; *Z_C_*], and we have the following equations:

(6) $$\begin{array}{l} x_{h}=\frac{Y_{C}}{Z_{C}}f_{x}\\ y_{h}=-\frac{X_{C}}{\left(Z_{C}+ad\right)}f_{y} \end{array}$$

Given Equations (3) and (6), the coordinates of the object point can be calculated as:

(7) $$\begin{array}{l} X_{C}=ad\frac{x_{v}y_{v}}{f_{y}x_{h}-f_{x}y_{v}}\\ Y_{C}=ad\frac{x_{h}y_{v}}{f_{y}x_{h}-f_{x}y_{v}}\\ Z_{C}=ad\frac{f_{x}y_{v}}{f_{y}x_{h}-f_{x}y_{v}} \end{array}$$

Thus, given the internal parameters *f_x_*, *f_y_*, and *ad*, as well as the image coordinates (*x_v_*, *y_v_*) and (*x_h_*, *y_h_*) of an object point in an ideal anamorphic lens, the 3D coordinates of this point can be easily reconstructed using Equation (7).

### 3.2. Point Matching

**Figure 3:**
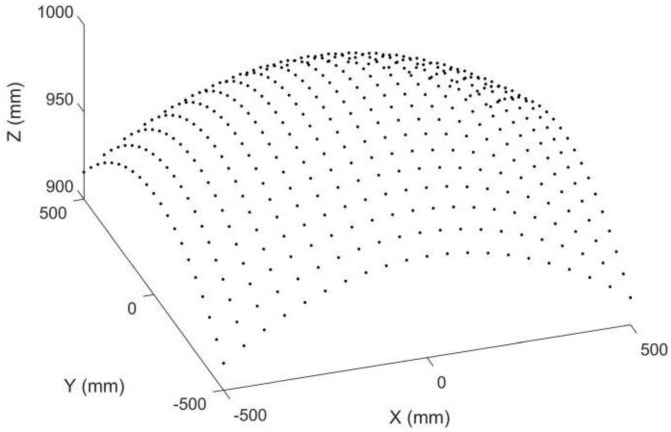
Simulated object points on a spherical surface in anamorphic coordinates of the vertical position.

**Figure 4:**
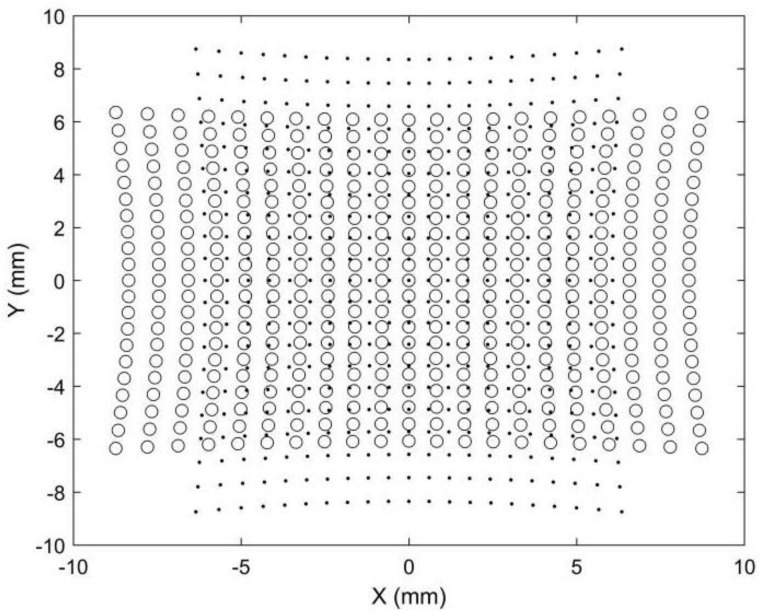
Simulated image points on the image plane. The dot points refer to the image points when the anamorphic lens is in the vertical position, and the circle points refer to the image points when the anamorphic lens is in the horizontal position.

**Figure 5:**
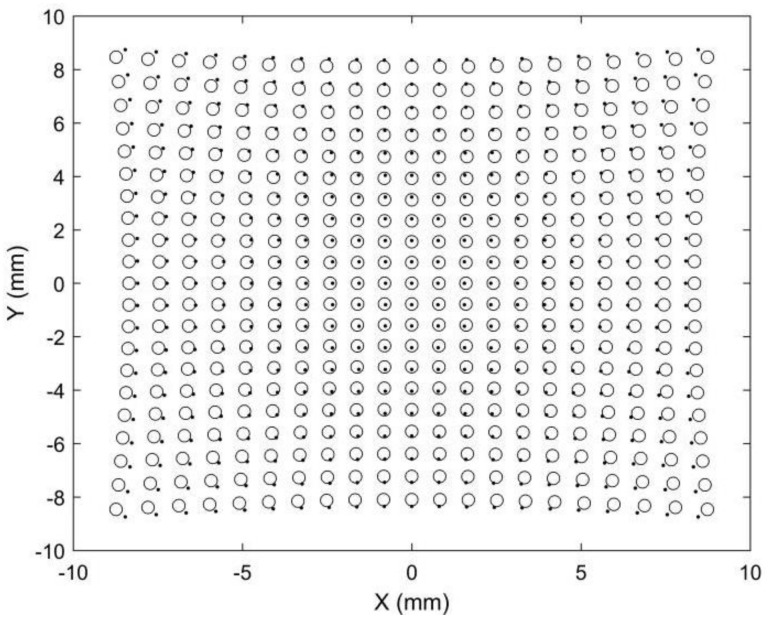
Simulated image points after anamorphic ration (*AR*) expansion. The dot points are rectified horizontally by *AR*, and the circle points are rectified vertically by *AR*.

**Figure 6:**
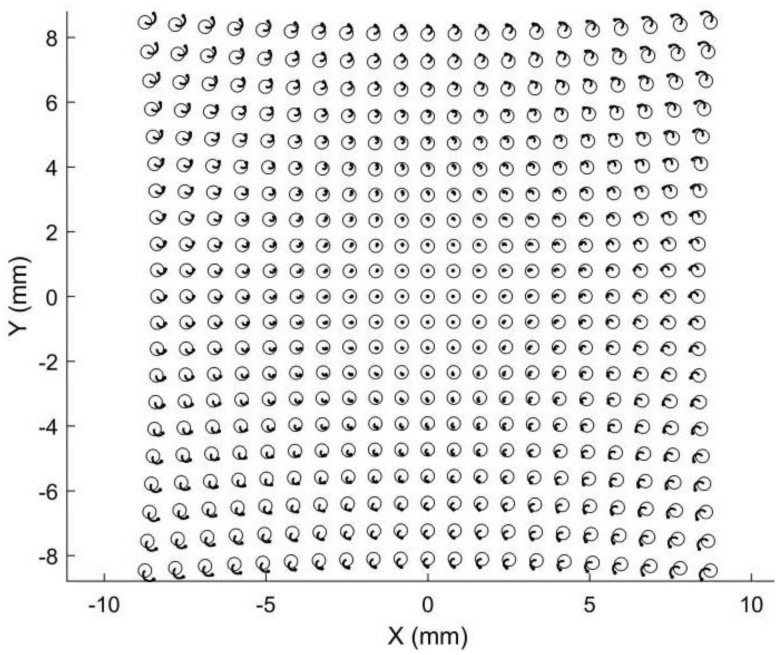
Simulated image points tracing from the vertical position to the horizontal position.

Point matching is crucial for stereo vision but is also challenging because the two images are captured by two different cameras. Points in different positions will have different image points in stereo images. Epipolar constraints and projectors are commonly adopted to achieve robust point matching, which requires a complex calculation. Point matching limits the applications of stereo vision in areas requiring fast or real-time 3D reconstruction, such as car navigation. Compared with stereo vision, point matching is much simpler in our proposed 3D metrology method. A simulation is provided to demonstrate its image point matching, as shown in Figure 3, Figure 4, Figure 5 and Figure 6. The simulated anamorphic lens has the following internal parameters: *ad* = 30 mm, *aa* = 0°, *f_x_* = 12 mm, *f_y_* = 16 mm, and no distortions. As shown in Figure 3, the object to be built is a collection of points on a spherical surface, with their coordinates given in the vertical position of the anamorphic lens. The simulated image points on the image planes are shown in Figure 4, where the dot points and the circle points express the image points when the anamorphic lens is in the vertical and horizontal positions, respectively. It is not easy to match the image points from these two positions in the current stage. In our method, the point matching can be greatly simplified by using a parameter of an anamorphic lens known as the anamorphic ratio *AR*, which is:

(8) $$AR\approx \frac{f_{y}}{f_{x}}$$

As a result, if the dot points and circle points in Figure 4 are expanded horizontally and vertically by *AR,* respectively, we obtain the corresponding image points shown in Figure 5. Because the corresponding points are nearby dot points and circle points, point matching in rotating anamorphic lenses becomes a simple and straightforward task. In addition, as illustrated in Figure 6, we can trace the image positions during the anamorphic rotation from the dot points to the circle points for point matching. Point matching in anamorphic rotating 3D metrology is much easier than stereo vision with spherical lenses, as shown in Figure 5 and Figure 6. The deviations between the dot points and the circle points are due to the anamorphic distance *ad*. In this example, the anamorphic distance of 30 mm was much smaller than the object distance of 1500 mm. The deviation between the dot points and the circles in the image planes will increase when the object points are imaged using an anamorphic lens with a large anamorphic distance.

### 3.3. Stereo Vision with Anamorphic Lenses

Inevitably, there will be a misalignment between the optical axis of the anamorphic lens and the rotation axis of the rotary table, which will be magnified if the object is far away from the lens. As a result, large errors will occur if Equation (7) is applied directly for 3D reconstruction. A rational approach for high-precision 3D metrology is to treat the rotating anamorphic lenses as two distinct anamorphic lenses, which constitute a stereo vision with anamorphic lenses. The main differences between stereo vision and stereo vision with anamorphic lenses are that spherical lenses are replaced by anamorphic lenses and the baseline distance between the two anamorphic lenses is extremely small.

For 3D metrology using rotating anamorphic lenses, we should first calibrate the internal parameters of the anamorphic lenses according to the anamorphic imaging model and the anamorphic distortion model. A 3D calibration target can be used to calibrate the anamorphic lens [16]. As can be seen in Equation (2), the distortion model of the anamorphic lens has 19 unknown distortion coefficients. If only one image of a 3D calibration target is used for anamorphic lens calibration, local convergence is likely to occur for the distortion coefficients. Commonly, the 3D calibration target cannot cover the entire imaging area, which will lead to a poor calibration result for other imaging areas. Therefore, to achieve a stable calibration result for the entire imaging area, mixed calibration targets are adopted, which means 3D calibration targets and 2D calibration targets are both required [25]. The initial values of the internal parameters are calibrated using the 3D calibration target, and those parameters are refined using 2D calibration targets. After anamorphic calibration using mixed calibration targets, the internal anamorphic lens parameters [*f_x_*, *f_y_*, *u*_0_, *v*_0_, *ad*, *aa*] and the 19 distortion coefficients [*k*_1_, *k*_2_, *n*_1_, *n*_2_, *m*_1_, *m*_2_, *x*_21_, *x*_12_, *x*_03_, *x*_20_, *x*_11_, *x*_02_, *y*_30_, *y*_21_, *y*_12_, *y*_03_, *y*_20_, *y*_11_, *y*_02_] can be determined.

After anamorphic lens calibration, we should calibrate the relative position of the anamorphic lens in the vertical position and the horizontal position. Two images of the 3D calibration target are captured, one for the vertical position and one for the horizontal position. Based on the calibrated internal parameters of the anamorphic lens and the image of the 3D calibration target, the relative position [*t_x_*, *t_y_*, *t_z_*, *a*, *b*, *r*] between the 3D calibration target and the anamorphic lens can be easily calibrated:

(9) $$T=\left[\begin{array}{c} t_{x}\\ t_{y}\\ t_{z} \end{array}\right]$$

(10) $$R=\left[\begin{array}{lll} \cos(b)\cos(r) & \sin(a)\sin(b)\cos(r)-\cos(a)\sin(r) & \sin(a)\sin(r)+\cos(a)\sin(b)\cos(r)\\ \cos(b)\sin(r) & \cos(a)\cos(r)+\sin(a)\sin(b)\sin(r) & \cos(a)\sin(b)\sin(r)-\sin(a)\cos(r)\\ -\sin(b) & \cos(b)\sin(a) & \cos(a)\cos(b) \end{array}\right]$$

where *T* and *R* refer to the translation vector and the rotation matrix. For the two anamorphic positions, there are the following equations between the coordinates of the 3D calibration target and the two anamorphic coordinates in the two positions:

(11) $$\begin{array}{l} P_{CV}=R_{CV}^{W}P_{W}+T_{CV}^{W}\\ P_{CH}=R_{CH}^{W}P_{W}+T_{CH}^{W} \end{array}$$

where the superscript *W* refers to the coordinates of the 3D calibration target, and the subscripts *CV* and *CH* refer to the coordinates of the anamorphic lens in the vertical and horizontal positions. Given Equation (11), the relationship between the vertical and the horizontal anamorphic coordinates can be determined as follows:

(12) $$P_{CH}=R_{CH}^{CV}P_{CV}+T_{CH}^{CV}$$

where $R_{CH}^{CV}$ and $T_{CH}^{CV}$ are determined as follows:

(13) $$\begin{array}{l} R_{CH}^{CV}=R_{CH}^{W}{\left(R_{CV}^{W}\right)}^{-1}\\ T_{CH}^{CV}=T_{CH}^{W}-R_{CH}^{CV}T_{CV}^{W} \end{array}$$

The horizontal position is rotated from the vertical position by −90° along the optical axis; thus, the relative position between the two anamorphic positions is similar to [*t_x_*, *t_y_*, *t_z_*, *a*, *b*, *r*] = [0 mm, 0 mm, 0 mm, 0°, 0°, 90°]. After the anamorphic lens calibration and the stereo calibration for the rotating anamorphic lenses, the 3D reconstruction using rotating anamorphic stereo vision is similar to that in stereo vision with spherical lenses.

## 4. Experiments

### 4.1. Experiments

**Figure 7:**
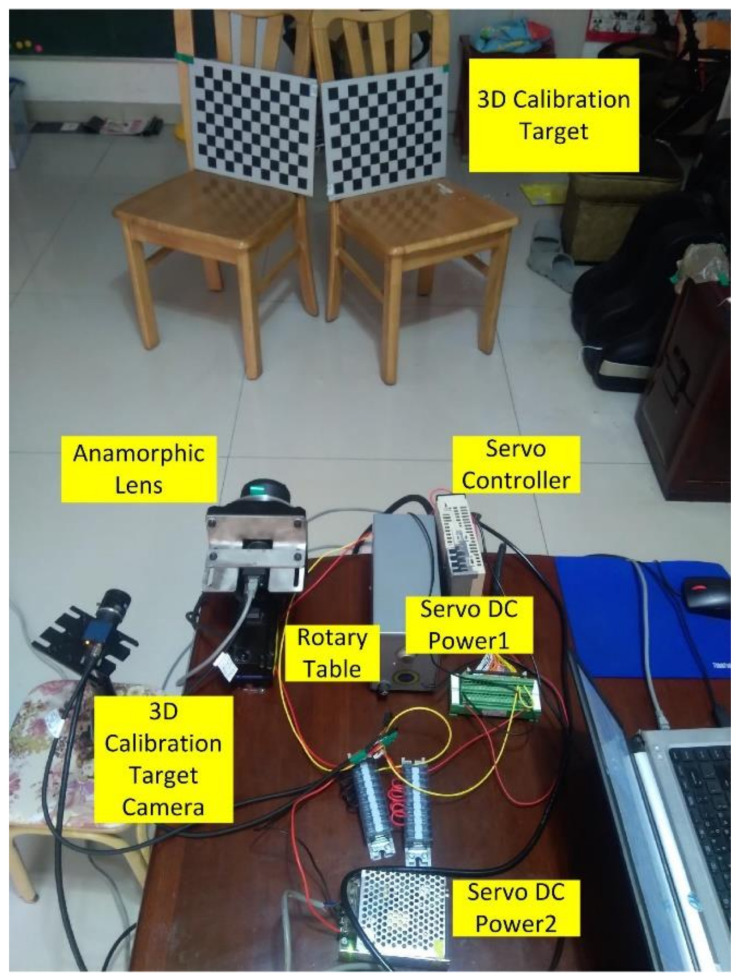
Experiment of 3D metrology using rotation anamorphic lenses.

**Figure 8:**
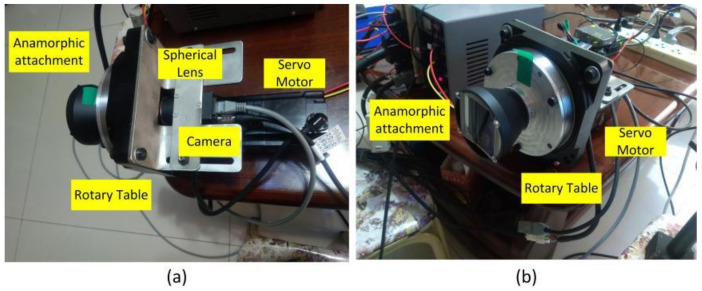
Anamorphic lens composed of a front anamorphic attachment and a rear spherical lens. The anamorphic lens was mounted on a rotary table which could rotate the anamorphic lens by −90° along the optical axis. (**a**) Side view; (**b**) Front view.

**Figure 9:**
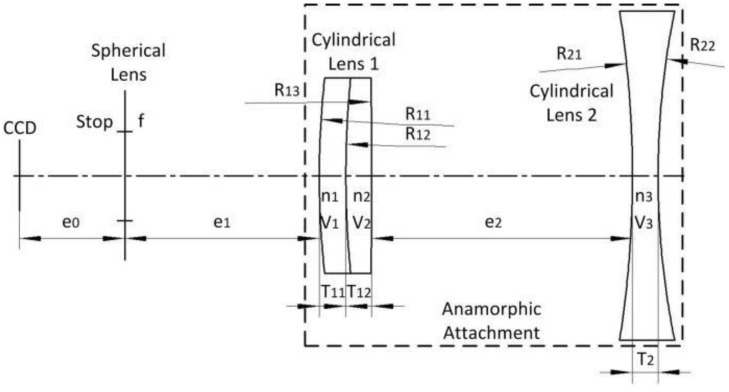
Anamorphic attachment for a Computar 16 mm spherical lens in the YOZ plane.

An experiment was performed to validate the proposed 3D metrology method using one camera with rotating anamorphic lenses. The design of the experiment is shown in Figure 7. The camera was MER-504-10Gx-P from Daheng China, and its resolution was 2448 × 2448 with a pixel size of 3.45 μm × 3.45 μm. The anamorphic lens was mounted on the rotary table HK130-10. The servo motor was SGM7J-02A7C6E, and the servo controller was SGD7S-1R6A00A002, both from YASKAWA, Japan. As shown in Figure 7, the 3D calibration target was composed of two 2D planar calibration targets, and the relative position of the two planar calibration targets was determined by the 3D calibration target camera, which was previously calibrated. As shown in Figure 8, the anamorphic lens was composed of a front anamorphic attachment and a 16 mm Computar spherical lens. As shown in Figure 9, the anamorphic attachment comprised three cylindrical lens elements, and the effective focal length in the YOZ plane was −2489 mm. A paraxial lens design for anamorphic lenses can be seen in [27,28]. The lens parameters for the anamorphic attachment are shown in Table 1.

**Figure 10:**
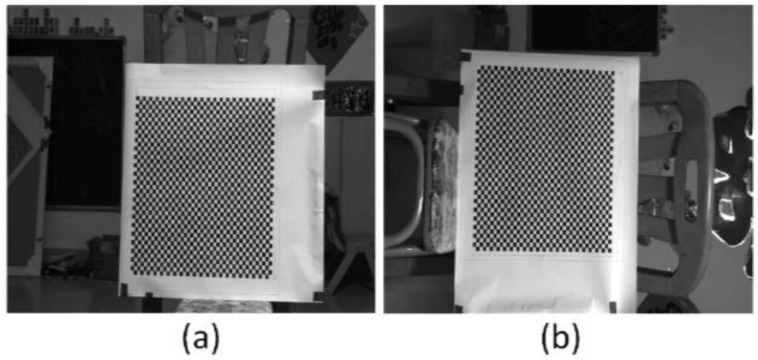
Anamorphic images. (**a**) Image when the anamorphic lens was in the vertical position, and (**b**) image when the anamorphic lens was in the horizontal position.

The anamorphic lens was calibrated using 3D and 2D calibration targets, and the internal parameters are shown in Table 2. And as shown in Figure 7, the relative position of the vertical position with respect to the horizontal position was calibrated using the 3D calibration target, and the results were [*t_x_*, *t_y_*, *t_z_*, *a*, *b*, *r*] = [−0.8943 mm, 0.3591 mm, −0.1712 mm, −0.5586°, −0.8074°, 90.0059°]. After the anamorphic lens calibration and the rotating anamorphic stereo calibration, we conducted the 3D metrology using the rotating anamorphic lens. As shown in Figure 10, to achieve dense 3D points, a checkerboard was adopted with a square’s length of 5 mm. The original anamorphic images are shown in Figure 10. Figure 10a shows the image when the anamorphic lens was in the vertical position, and Figure 10b shows the image when the anamorphic lens was in the horizontal position. Figure 10b was achieved by rotating the anamorphic lens by −90° anticlockwise along the optical axis. Once the corresponding corners in Figure 10a,b were determined, the 3D coordinates of the corners could be reconstructed from the rotating anamorphic stereo vision.

**Figure 11:**
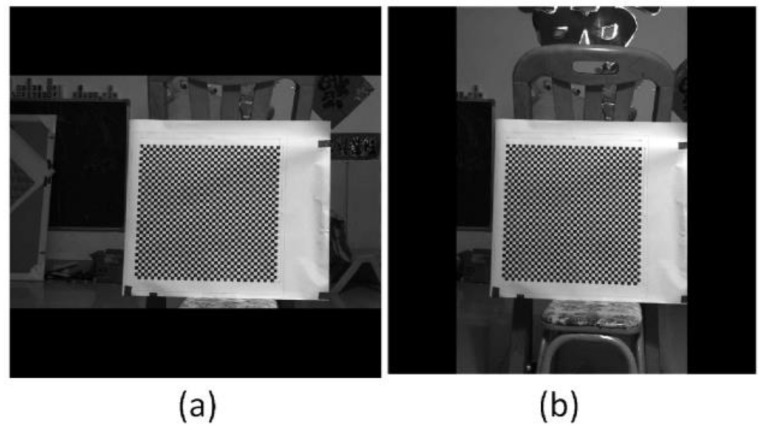
Anamorphic images after anamorphic ratio (*AR*) rectification. (**a**) Rectified image when the anamorphic lens was in the vertical position, and (**b**) rectified image when the anamorphic lens was in the horizontal position.

One possible method to determine the corresponding corners between Figure 10a,b is to trace the corners while rotating the anamorphic lens, similar to Figure 6. In order to trace the corners correctly, the rotating speed must be limited. Another method is to rectify the two anamorphic images by the anamorphic ratio *AR,* as shown in Equation (6), after which the corresponding points are very close, as in Figure 5. Figure 11a was obtained by expanding Figure 10a horizontally by the *AR*. Rotating Figure 10b by −90° anticlockwise and expanding the height of the rotated image by the *AR*, we obtained Figure 11b. As can be seen in Figure 11a,b, the two images are very similar after *AR* rectification, and the corresponding points are nearby, for the overlapping areas in Figure 11a,b.

**Figure 12:**
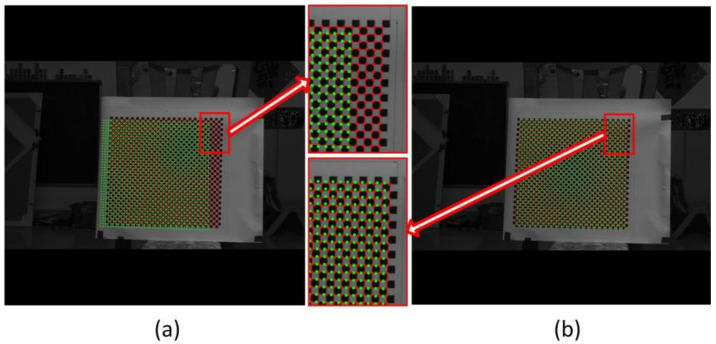
Corners after anamorphic ratio (*AR*) rectification. The red points indicate the corners in Figure 11a, and the green points indicate the corners in Figure 11b. (**a**) Image points in their original position, (**b**) green points shift their positions entirely, after which the two images almost overlap. This pixel decentering was due to the deviation between the optical axis and the axis of the rotary table.

The chessboard corners in Figure 11a were extracted and represented as red points in Figure 12; the corners in Figure 11b were represented by green circles in Figure 12. The red points and green circles in Figure 12a are in their original positions. It can be seen that the corresponding corners were not adjacent and were separated from one another. This is because there was a misalignment between the optical axis and the rotation axis of the rotary table. This misalignment was further amplified by the large object distance. If Equation (7) is directly applied for 3D reconstruction, there will be significant errors. Thus, the rotating anamorphic stereo vision was adopted for the 3D reconstruction. As can be seen in Figure 11, the two images are similar, the only difference between Figure 11a,b being a decentering. If we shift Figure 11b by *(d_u_*, *d_v_)* = (105.0792 pixels, −28.8297 pixels), the two images will overlap. In Figure 12b, the corresponding points are very close, as shown in Figure 5.

**Figure 13:**
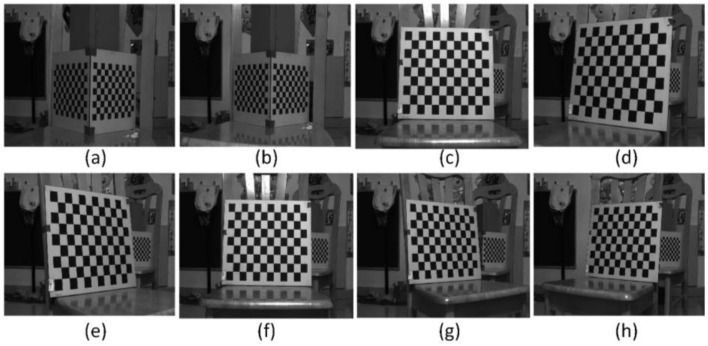
Images for 3D construction when the anamorphic lens was in the vertical position. (**a**,**b**) refer to images of 3D targets and (**c**–**h**) refer to the images of a 2D target.

**Figure 14:**
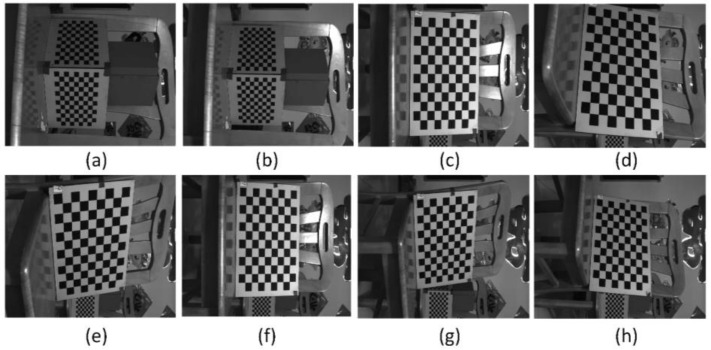
Images for 3D construction when the anamorphic lens was in the horizontal position. (**a**,**b**) refer to images of 3D targets and (**c**–**h**) refer to the images of a 2D target.

**Figure 15:**
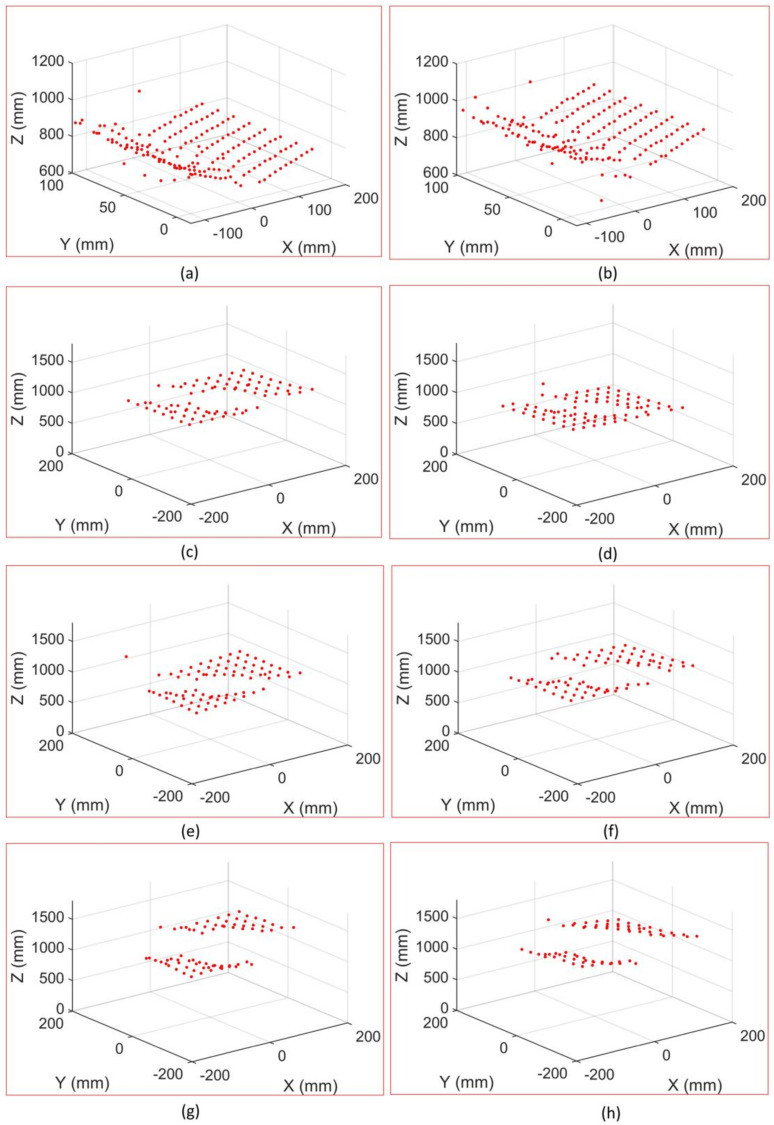
Constructed 3D points for images (**a**–**h**) in Figure 13 and Figure 14.

Given the internal parameters of the anamorphic lens, the relative positions between the vertical anamorphic lens and the horizontal anamorphic lens, and the corresponding image points, the 3D coordinates of the corners could be reconstructed using the rotating anamorphic stereo vision. As shown in Figure 13 and Figure 14, the 3D positions of the corners in checkerboards were constructed. Figure 13 shows the images of the checkerboards when the anamorphic lens was in the vertical position, and Figure 14 shows the images of the calibration targets when the anamorphic lens was in the horizontal position. When constructing the eight objects, the first two objects were 3D objects, and the other six objects were 2D objects. The constructed 3D points are shown in Figure 15, while not all corners in the checkerboard were reconstructed. Some points were no constructed for they lay in the anamorphic gas where the construction accuracy was very low. The anamorphic gas is discussed in detail in Section 4.2.2. As can be seen in Figure 15, the reconstruction accuracy was low compared with that of the stereo vision. The accuracy of this metrology method is also discussed in Section 4.2.

### 4.2. Accuracy Analysis

As shown in Equation (14), the pixel error was the main source of error for this metrology, though other parameters such as *ad*, *f_x_*, and *f_y_* are also very important. This method’s measuring principle is based primarily on the anamorphic distance, which changes the image position compared to spherical lenses. The anamorphic distance *ad* shown in Section 4.1 was small in comparison to the object distance; thus, any small pixel position error would have a significant impact on the measurement results. From Equation (7), we have:

(14) $$\begin{array}{l} dX_{C}=\frac{\partial X_{C}}{\partial x_{h}}\delta x_{h}+\frac{\partial X_{C}}{\partial x_{v}}\delta x_{v}+\frac{\partial X_{C}}{\partial y_{v}}\delta y_{v}\\ dY_{C}=\frac{\partial Y_{C}}{\partial x_{h}}\delta x_{h}+\frac{\partial Y_{C}}{\partial y_{v}}\delta y_{v}\\ dZ_{C}=\frac{\partial Z_{C}}{\partial x_{h}}\delta x_{h}+\frac{\partial Z_{C}}{\partial y_{v}}\delta y_{v} \end{array}$$

where *δx_h_*, *δx_v_*, and *δy_v_* are the pixel errors. If we substitute the pixel errors in Equation (14) with *δ*, the point error can be deduced from Equation (14) as follows:

(15) $$\begin{array}{l} \Delta xyz=\sqrt{dX_{C}{}^{2}+dY_{C}{}^{2}+dZ_{C}{}^{2}}\\ =ad\cdot \delta \cdot \frac{\sqrt{x_{2}y_{2}\cdot f_{x}^{2}\cdot f_{y}^{2}+x_{2}\cdot f_{x}^{2}+x_{1}y_{1}\cdot f_{x}\cdot f_{y}+y_{2}\cdot f_{y}^{2}}}{{\left(f_{y}\cdot x_{h}-f_{x}\cdot y_{v}\right)}^{2}} \end{array}$$

where the coefficients *x_2_y_2_*, *x_2_*, *x_1_y_1_*, and *y_2_* in Equation (15) are given by:

(16) $$\begin{array}{l} x_{2}y_{2}={\left(x_{h}-y_{v}\right)}^{2}\\ x_{2}=2y_{v}^{4}\\ x_{1}y_{1}=-2y_{v}^{2}\left(x_{h}^{2}+\left(x_{v}+y_{v}\right)x_{h}-x_{v}y_{v}\right)\\ y_{2}=x_{h}^{4}+{\left(x_{v}+y_{v}\right)}^{2}x_{h}^{2}-2x_{v}y_{v}\left(x_{v}+y_{v}\right)x_{h}+x_{v}^{2}y_{v}^{2} \end{array}$$

To further evaluate the measurement accuracy, we conducted two simulation experiments. The simulation anamorphic lens was the same as the anamorphic lens shown in Section 3.2: *f_x_* = 12 mm, *f_y_* = 16 mm, *aa* = 0°, and *ad* = 30 mm.

#### 4.2.1. Accuracy Analysis for a Point

**Figure 16:**
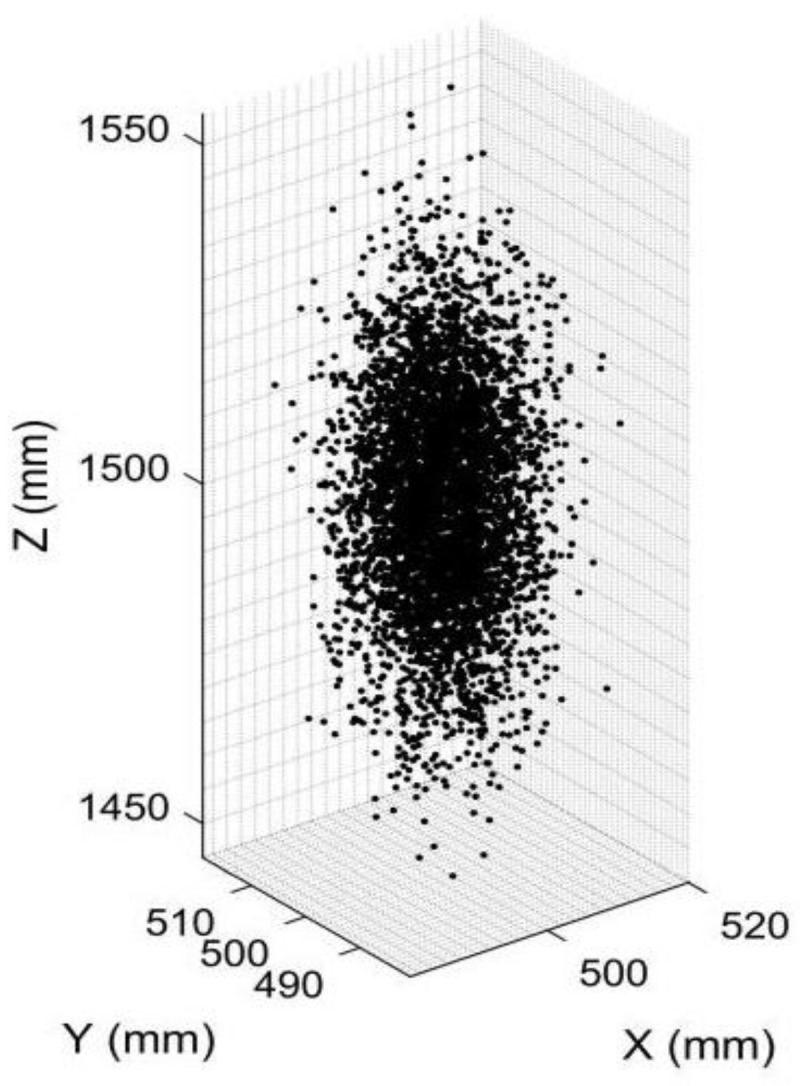
Image showing 5000 constructed 3D points for an object point in [500 mm, 500 mm, 1500 mm] with different pixel errors. The standard deviation of the constructed 3D points was 17.3378 mm.

First, we simulated an object point with the coordinates [*X_C_, Y_C_, Z_C_*] = [500 mm, 500 mm, 1500 mm] in anamorphic coordinates for the vertical position. The pixel coordinates of the point in the vertical and the horizontal anamorphic positions were calculated using Equations (3)–(6). Given the pixel coordinates and the calibrated parameters of the rotating anamorphic stereo vision, the 3D coordinates of the point could be calculated. The pixel errors in the image plane were assumed to have a Gaussian normal distribution with a standard deviation of 0.2 pixels, which was the calibration result of the anamorphic lens. Then, 5000 3D points were reconstructed with varying pixel errors, and the reconstructed 3D points are shown in Figure 16. The standard deviation of the reconstructed 3D point was 17.3378 mm.

#### 4.2.2. Accuracy Analysis for a Surface

**Figure 17:**
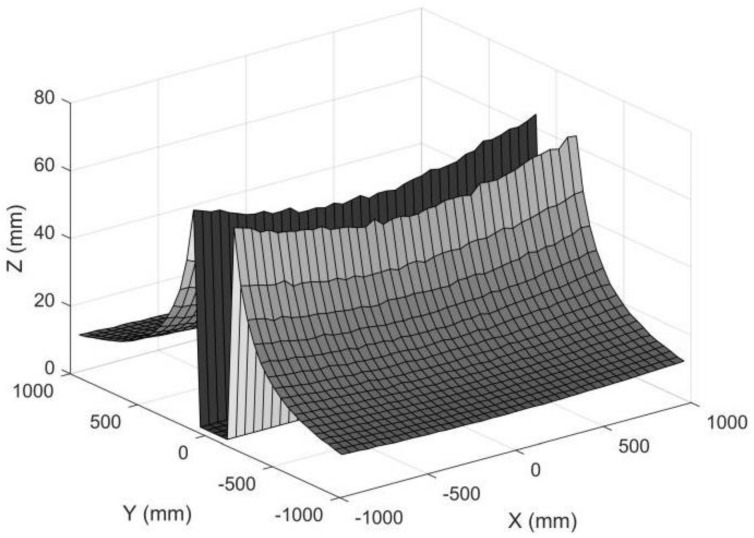
Standard deviation for object points in a plane. The standard deviations for object points with small *Y_C_* coordinates were removed for large reconstruction errors. The standard deviation of the pixel position was 0.2 pixels, the ad was 30 mm, and the *Z_C_* was 1500 mm.

We also simulated a set of object points in a plane. The *X_C_* and *Y_C_* coordinates ranged from −1000 mm to 1000 mm, with an interval of 50 mm, and all the *Z_C_* were 1500 mm. The pixel errors in the image plane satisfied a Gauss normal distribution, and the standard deviation of the pixel error was set to 0.2 pixels. The standard deviation for each reconstructed 3D point is shown in Figure 16. As can be seen in Figure 17, the measuring accuracy for the points away from the *X_C_* axis was high, while it was very low if the object points were near the *X_C_* axis. The points near the *X_C_* axis were removed, which left a gap in Figure 17. Thus, there was a measuring gap if rotating anamorphic lenses were used for 3D construction. We named this gap the anamorphic gap.

**Figure 18:**
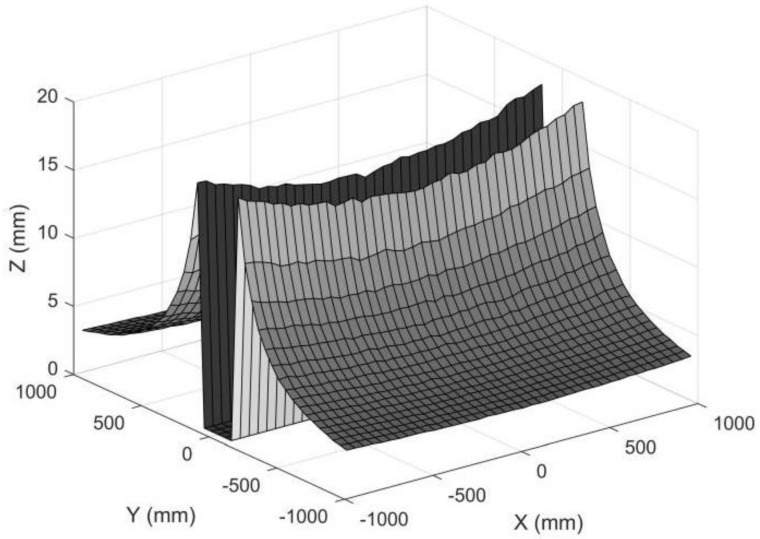
Standard deviation for object points in a plane. The standard deviations for object points with small *Y_C_* coordinates were removed for large reconstruction errors. The standard deviation of the pixel position was 0.2 pixels, the ad was 100 mm, and the *Z_C_* was 1500 mm.

If we individually increase the anamorphic distance from 30 mm to 100 mm, the measuring accuracy will increase correspondingly, as shown in Figure 18. The lens parameters *ad*, *f_x_*, and *f_y_* are closely related because they are determined by the anamorphic lens structure, and it is not possible to simply change one parameter independently [25]. We proposed paraxial lens designs for anamorphic lenses with zero anamorphic distance [26], but designing an anamorphic attachment with an extremely large anamorphic distance appeared to be difficult.

The measuring precision of this 3D metrology method appeared to be lower than that of stereo vision with spherical lenses. The fundamental reason for this is that, in comparison to stereo vision, the baseline distance between the two anamorphic positions was quite small. The measuring precision will be as good as that of stereo vision with spherical lenses if the two anamorphic lenses are sufficiently apart from each other. Because the two images are captured with the same revolving anamorphic lens, the main advantage of this 3D metrology is its ease of point matching. The appropriate matching points are very close if the two images are rectified with the anamorphic ratio, as shown in Figure 5 and Figure 12. Alternatively, as illustrated in Figure 6, it is possible to trace the matching points by rotating the anamorphic lens. When the anamorphic lens is rotated at a rapid speed, it can measure 3D data four times in one circle, which is ideal for a quick 3D reconstruction with low measurement accuracy. One potential application of this would be car navigation.

## 5. Conclusions

A 3D metrology method using one camera with rotating anamorphic lenses is proposed in this paper. The anamorphic lens can rotate by −90° along the optical axis, capturing two anamorphic images of an object before and after the rotation. This setup is similar to that of stereo vision; therefore, it can be considered a stereo vision with anamorphic lenses. The main differences between stereo vision and rotating anamorphic lenses are that anamorphic lenses replace spherical lenses, and the baseline distance between the two anamorphic lenses is very small. This feature will make rotating anamorphic lenses a compact sensor suitable for 3D metrology in a constrained space. The feasibility of the proposed method in 3D reconstruction is based on one internal parameter of anamorphic lenses, i.e., the anamorphic distance, which will slightly change the image position of the object. The corresponding points between the two anamorphic images are close if the two anamorphic images are rectified by the anamorphic ratio, which substantially simplifies the corresponding point matching and reduces the calculation amount. When compared to stereo vision, the measurement accuracy of the rotating anamorphic stereo vision is lower, especially for the image points near the *X_C_* axis of the anamorphic lens in the vertical position. These characteristics make the rotating anamorphic stereo vision suitable for a fast 3D reconstruction without a high demand for measurement accuracy, such as car navigation applications. Further research might include high precision anamorphic lens calibration, error compensation, point matching for rotating anamorphic lenses, and anamorphic lens designs with a large anamorphic distance.

## Figures and Tables

**Table 1:** Parameters of the anamorphic attachment used for the anamorphic lens.

*e*_0_ (mm)	*e*_1_ (mm)	*e*_2_ (mm)	*T*_11_ (mm)	*T*_12_ (mm)	*T*_2_ (mm)
16.163062	30	40	4	4	4
*f* (mm)	*R*_11_ (mm)	*R*_12_ (mm)	*R*_13_ (mm)	*R_21_* (mm)	*R*_22_ (mm)
16	155	156.4	−857.6	−174.8	124.5
*n*_1_	*V*_1_	*n*_2_	*V*_2_	*n*_3_	*V*_3_
1.516797	64.212351	1.672702	32.17888	1.516797	64.212351

**Table 2:** Internal parameters of the calibrated anamorphic lens.

*f_x_* (mm)	*f_y_* (mm)	*u*_0_ (pixel)	*v*_0_ (pixel)	*ad* (mm)
12.0520	16.1026	1.2790 × 10^3^	1.0023 × 10^3^	26.5516
*aa* (°)	*k*_1_	*k*_2_	*n*_1_	*n*_2_
−0.5789	0.0304	−2.7151 × 10^−5^	0.0183	−3.3372 × 10^−5^
*m*_1_	*m*_2_	*x*_21_	*x*_12_	*x*_03_
−0.0155	1.277e-5	9.1986 × 10^−5^	−0.0139	1.8928 × 10^−5^
*y*_30_	*y*_21_	*y*_12_	*y*_03_	*x*_20_
1.6727 × 10^−5^	0.011	1.0611 × 10^−4^	−0.0023	1.3802 × 10^−4^
*x*_11_	*x*_02_	*y*_20_	*y*_11_	*y*_02_
−4.7071 × 10^−5^	5.8709 × 10^−5^	9.3657 × 10^−5^	−3.9944 × 10^−4^	−2.9851 × 10^−4^

## Data Availability

The data underlying the results presented in this paper are not publicly available at this time but may be obtained from the authors upon reasonable request.
